# Calorie Restriction Leads to Degradation of Mutant Uromodulin and Ameliorates Inflammation and Fibrosis in *UMOD*-Related Kidney Disease

**DOI:** 10.1681/ASN.0000001032

**Published:** 2026-02-03

**Authors:** Mariapia Giuditta Cratere, Benedetta Perrone, Barbara Canciani, Céline Schaeffer, Luca Rampoldi

**Affiliations:** 1 Molecular Genetics of Renal Disorders, Division of Genetics and Cell Biology, IRCCS San Raffaele Scientific Institute, Milan, Italy; 2 Vita-Salute San Raffaele University, Milan, Italy; 3 IRCCS Ospedale Galeazzi-Sant’Ambrogio, Milan, Italy

**Keywords:** CKD, endoplasmic reticulum, interstitial fibrosis, malfolding proteins, molecular genetics, progression of renal failure, transgenic mouse, tubulointerstitial disease, genetic kidney disease, pathophysiology

## Abstract

**Key Points:**

- Calorie restriction stimulated autophagy and degradation of mutant uromodulin, leading to amelioration of cell stress and tubular damage.
- At early disease stage, calorie restriction largely reverted autosomal dominant tubulointerstitial kidney disease (ADTKD)-*UMOD* phenotype, preventing inflammation, fibrosis, and kidney function decline.
- At advanced disease stage, calorie restriction significantly delayed disease progression and worsening of kidney function.

**Background:**

Mutations in *UMOD*, encoding uromodulin, lead to autosomal dominant tubulointerstitial kidney disease (ADTKD), a genetic cause of kidney failure. *UMOD* mutations have a common gain-of-toxic-function effect, causing mutant uromodulin retention in the endoplasmic reticulum (ER). This leads to ER stress, alteration of protein homeostasis and mitochondrial dynamics, defective autophagy, and increased cell death. Calorie restriction exerts a beneficial role in diseases characterized by accumulation of pathogenic protein and inflammation, by modulating several pathways, including autophagy induction and suppression of inflammation and fibrosis. Given the relevance of these features in ADTKD, we investigated the effect of calorie restriction on disease onset and progression.

**Methods:**

Transgenic mice expressing C147W uromodulin (Tg^*Umod*C147W^) were subjected to a moderate (30%) calorie restriction regimen for 15 or 24 weeks, starting at different stages of disease progression.

**Results:**

Calorie restriction restored autophagy, as shown by decreased P62 punctae and quenched mammalian target of rapamycin (mTOR) activation specifically in mutant uromodulin-expressing cells, and it recovered expression of key ER-phagy receptor genes, with a concomitant, striking reduction of mutant uromodulin ER retention. In presymptomatic Tg^*Umod*C147W^ mice, calorie restriction alleviated epithelial cell stress. This, likely along with a direct anti-inflammatory effect of calorie restriction, prevented inflammation and progressive decline of kidney function. At this early disease stage, calorie restriction ameliorated the already established kidney damage and reduced fibrosis, suggesting reversal of ADTKD phenotype. Calorie restriction was also effective in significantly delaying disease progression in Tg^*Umod*C147W^ mice with advanced disease and already compromised kidney function.

**Conclusions:**

Calorie restriction enhanced autophagy and uromodulin degradation, counteracting the primary effect of *UMOD* mutations, and significantly ameliorated kidney disease onset and progression.

## Visual Abstract

**Figure:**
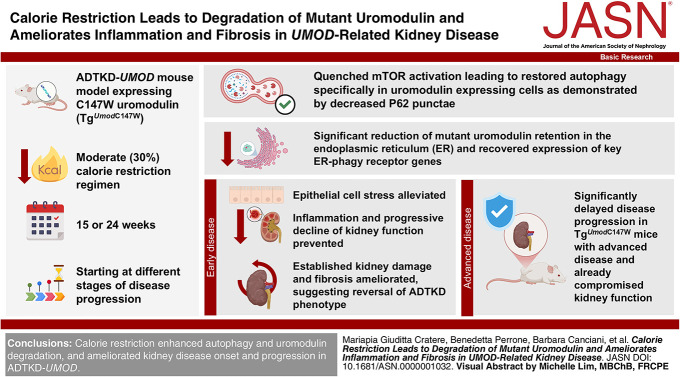

## Introduction

*UMOD*-related autosomal dominant tubulointerstitial kidney disease (ADTKD-*UMOD*; MIM #16200) is a Mendelian disorder characterized by tubulointerstitial fibrosis, urine concentration defect, hyperuricemia, and progressive loss of kidney function leading to kidney failure mainly between 20 and 50 years.^1,2^ ADTKD-*UMOD* is caused by mutations in the *UMOD* gene^3^ and represents the third most frequent kidney monogenic disorder,^4^ accounting for 2% of all kidney failure cases.^5^

*UMOD* encodes uromodulin,^6,7^ the major urinary protein specifically produced by tubular epithelial cells (TECs) lining the thick ascending limb of Henle's loop (TAL) and the early distal convoluted tubules.^8^ *UMOD* mutations are mostly missense,^9,10^ causing misfolding of uromodulin and its accumulation in the endoplasmic reticulum (ER), inducing ER stress and the unfolded protein response. These features are observed in patient samples^11–14^ and recapitulated in several *in vitro*^15–18^ and *in vivo* models,^11,14,19–22^ ascribing ADTKD-*UMOD* as an ER storage disease.^23^ The different extent of ER retention associates with disease severity, and it was proposed as a predictor of age at kidney failure.^9^ Moreover, propensity of mutant uromodulin to aggregate contributes to disease pathogenesis and correlates with disease severity.^14^ Additional disease features are altered mitochondrial dynamics,^21,22^ defective protein homeostasis, and impaired autophagy in TAL cells.^14,19,22^ Inflammation is a key early pathogenetic event, preceding fibrosis and occurring concomitantly with TAL cell stress.^24^

An effective, specific therapeutic strategy is presently an unmet clinical need. Given the gain-of-(toxic)-function effect of *UMOD* mutations, removal of intracellularly retained mutant protein could be a valuable option. This is supported by recent evidence showing that promoting mutant uromodulin trafficking and secretion reduced kidney damage in an *in vivo* disease model.^25^ Similarly, enhancing autophagy may be beneficial, as supported by evidence that autophagy stimulation promotes degradation of uromodulin aggregates in cell models^14^ and ameliorates phenotype in ADTKD-*UMOD* mouse models.^22^

Calorie restriction, consisting in reducing daily calorie intake by 20%–50% without causing malnutrition,^26^ is beneficial in different settings characterized by accumulation of pathogenic proteins and inflammation.^27,28^ Its protective role is exerted through several mechanisms, including improvement of mitochondrial and autophagy dysfunction and suppression of inflammation and oxidative stress.^29^

Since these are all key features of the disease,^14,19,21,22,24^ here we investigated the therapeutic potential of calorie restriction in ADTKD-*UMOD.* To this end, transgenic mice expressing C147W mutant uromodulin and recapitulating the human disease^11,24^ were subjected to a moderate 30% calorie restriction regimen^30,31^ for 15 or 24 weeks starting at early or advanced stages of disease progression. We aimed at testing if calorie restriction could revert the already established kidney damage and counteract disease progression. Moreover, we sought to assess the effect of the dietary intervention on uromodulin-expressing cells, by assessing uromodulin ER retention, autophagy induction, and cell damage.

## Methods

### Animal Models

All animals used in this study were housed under specific pathogen-free conditions with controlled humidity and temperature on a 12-hour dark/light cycle and *ad libitum* (AL) access to tap water and standard chow (Diet VRF1 [P], Special Diets Services, Augy, France), except during periods of calorie restriction experiments (see below). All procedures were reviewed and approved by the Institutional Animal Care and Use Committee of the San Raffaele Institute and were in accordance with the institutional guidelines (protocol #1368, #1526). Transgenic mouse lines expressing mutant (Tg^*Umod*C147W^) or wild-type (Tg^*Umod*wt^) uromodulin (*i.e*., serving as an expression matched control), both on the FVB/NJ background, were previously generated and characterized.^11,24^ Sequences of primer sets used for genotyping are listed in Supplemental Table 1.

### Calorie Restriction Protocols

Calorie restriction was started at 8 weeks (early disease stage) for 15 weeks or at 24 weeks (late disease stage) for 24 weeks. Experimental animals were randomly divided into AL or calorie-restricted groups and individually housed to measure the exact food intake. Food consumption in the AL groups was determined by weighting leftover chow daily. Animals in the calorie-restricted groups received 70% of the food consumed by mice in the AL regimen, as previously reported.^32–34^ Body weight of animals was assessed weekly. For all experiments, age-matched male and female mice were used. The number of mice in each experiment is reported in the figure legends. Data were analyzed separately for females and males for the protocol at early disease stage, while they were combined for the advanced stage one (see Supplemental Methods for additional details).

### Statistical Analysis

Data are reported as mean±SD or SEM. Statistical analysis was performed with Prism V10.4.2 software (GraphPad Inc.). The unpaired Student's *t* test was performed when comparing mice of the same genotype subjected to different diets. Comparison between more than two experimental groups was performed by using one- or two-way ANOVA followed by Dunnett, Šídák, or Tukey correction as indicated in the figure legends. A *P* < 0.05 was considered statistically significant. Multivariable data analysis was performed using unsupervised principal component analysis (PCA) to integrate all parameters tested. Data were normalized by autoscaling transformation to ensure comparability across variables measured with different scales. Experimental group clusters were visualized with 95% confidence interval ellipses that were automatically generated by the software. To evaluate the statistical significance of group separation, Permutational Multivariate Analysis of Variance (PERMANOVA) was performed. PCA, heatmaps of disease parameters, and K-means clustering analyses were generated using MetaboAnalyst 6.0. online package (www.metaboanalyst.ca/home.xhtml).

### Additional Methods

Detailed methods are presented in Supplemental Methods and Supplemental Tables 1, 10, and 11.

## Results

### Calorie Restriction Reduced Mutant Uromodulin ER Retention and Aggregation

**Figure 1:**
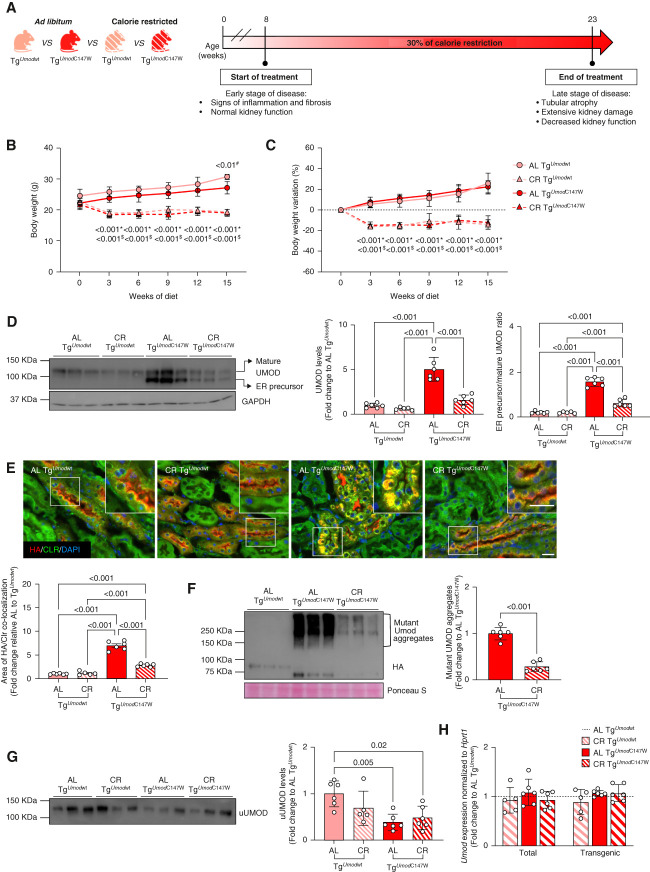
**Calorie restriction reduced mutant uromodulin ER retention and aggregation.** (A) Graphical representation of the calorie restriction protocol. (B and C) Body weight (B) and percentage of weight loss compared with the initial time point (C) of mice during the diet. ^#^*P* AL Tg^*Umod*wt^ versus AL Tg^*Umod*C147W^; ^*^P AL Tg^*Umod*wt^ versus calorie restriction Tg^*Umod*wt^; ^$^*P* AL Tg^*Umod*C147W^ versus calorie restriction Tg^*Umod*C147W^. (D) Western blot analysis of UMOD in kidney lysates. The ER precursor (immature, 90 kDa) and mature form of UMOD (120 kDa) are indicated. GAPDH is reported as a normalizer. Graphs represent quantification of total UMOD kidney levels and the ratio of ER precursor to mature UMOD isoforms. (E) Immunofluorescence analysis of transgenic UMOD (HA, in red) and the ER marker calreticulin (CLR, in green). Nuclei are counterstained with DAPI (blue). The magnified field is indicated by a square on each picture. Scale bar: 20 *μ*m. Histograms represent the quantification of the area of transgenic UMOD-CLR colocalization. (F) Western blot in nonreducing conditions of transgenic UMOD (anti-HA). HMW signal corresponds to mutant UMOD aggregates. Ponceau S staining is shown as a loading control. Histograms represent the quantification of UMOD aggregates in kidneys from AL or calorie restriction Tg^*Umod*C147W^ mice. (G) Western blot and quantification of uUMOD levels. Urine samples were normalized over total volume of 16-hour urine collection in metabolic cage. (H) Total and transgenic *Umod* transcript levels measured by real-time RT-qPCR in kidneys from AL and calorie restriction Tg^*Umod*wt^ and Tg^*Umod*C147W^ mice. *n*=5–6 mice/group (all females). Data are reported as fold change relative to AL Tg^*Umod*wt^ (in D, E, G, and H) or AL Tg^*Umod*C147W^ (in F). Bars represent mean±SD (in B–D, F, G, and H) or mean±SEM (in E). Group comparisons were performed by one-way ANOVA, followed by Šidák (in B and C) or Tukey (in D, E, and G) corrections or unpaired *t* test (in F). AL, *ad libitum*; CR, calorie restriction; DAPI, 4′,6-diamidino-2-phenylindole; ER, endoplasmic reticulum; GAPDH, glyceraldehyde 3-phosphate dehydrogenase; HA, hemagglutinin; HMW, high molecular weight; RT-qPCR, reverse transcriptase-quantitative PCR; Tg^*Umod*C147W^, transgenic mice expressing mutant (C147W) uromodulin; Tg^*Umod*wt^, transgenic mice expressing wild-type uromodulin; uUMOD, urinary uromodulin.

To test the hypothesis that reducing calorie intake could decrease mutant uromodulin intracellular load, we performed a 15-week long, 30% calorie restriction experiment in Tg^*Umod*C147W^ and Tg^*Umod*wt^ mice^11^ (Figure 1 for females, Supplemental Figure 1 for males). The initial time point (8 weeks) corresponds to an early phase of disease, with signs of kidney inflammation and fibrosis but preserved kidney function.^24^ At the final time point (23 weeks), Tg^*Umod*C147W^ mice show the full-blown disease, with reduced kidney function^11^ (Figure 1A and Supplemental Figure 1A).

Body weight of mice under calorie restriction decreased (<20% of the initial body weight) during the first 3 weeks, and it then stabilized until the end of treatment (Figure 1, B and C, and Supplemental Figure 1, B and C). Compared with Tg^*Umod*wt^, AL Tg^*Umod*C147W^ mice showed uromodulin accumulation within the kidney, which was massively decreased by calorie restriction (Figure 1D and Supplemental Figure 1D). Such reduction was mostly contributed by a significant decrease of uromodulin ER precursor (*i.e*., uromodulin carrying ER-type glycans),^11^ reflecting reduced ER retention of mutant protein after calorie restriction (Figure 1D and Supplemental Figure 1D). This was confirmed by immunofluorescence staining, specifically analyzing the distribution of transgenic uromodulin and showing decreased colocalization of mutant protein with the ER chaperone calreticulin (Figure 1E and Supplemental Figure 1E). Such effect was associated with a strong decrease of mutant uromodulin aggregates, as shown by decreased level of high molecular weight forms of uromodulin (Figure 1F and Supplemental Figure 1F). As previously reported and consistent with impaired protein maturation, AL Tg^*Umod*C147W^ mice showed reduced urinary uromodulin levels. After calorie restriction, reduction of uromodulin levels in the kidney was not accompanied by increased urinary excretion (Figure 1G and Supplemental Figure 1G). Such effect was not due to changes of gene expression, as shown by comparable transcript levels of total and transgenic *Umod* in all experimental groups (Figure 1H and Supplemental Figure 1H).

Altogether, these results demonstrate that calorie restriction is effective in reducing mutant uromodulin ER retention and aggregation, without altering its expression, trafficking, or urinary secretion, irrespective of sex.

### Calorie Restriction Ameliorated ADTKD-*UMOD* Kidney Phenotype

**Figure 2:**
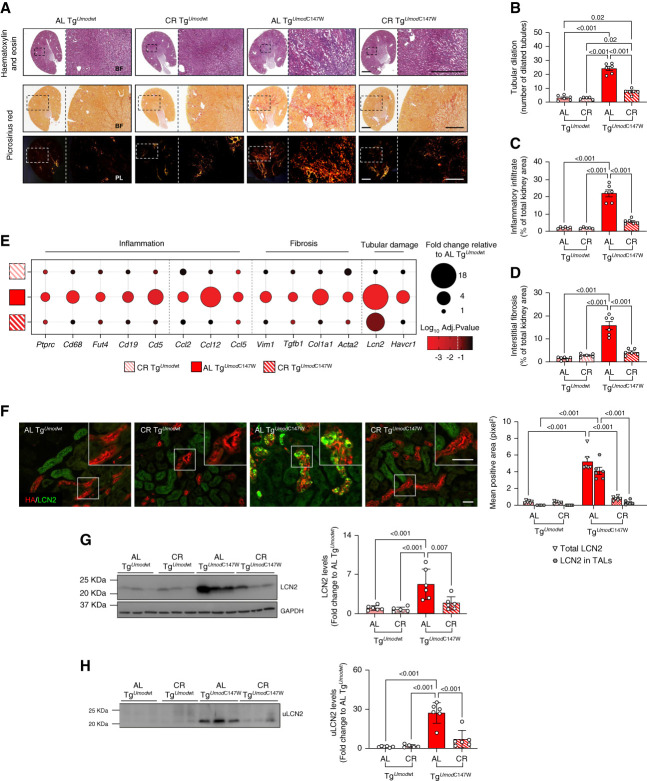
**Calorie restriction ameliorated kidney inflammation, fibrosis, and tubular damage.** (A) Representative histology images of kidney sections stained with hematoxylin and eosin or picrosirius red and viewed in bright field or under polarized light. Scale bars: 2 mm and 600 *μ*m. (B–D) Graphs represent quantification of tubular dilation (B) and areas of inflammatory cell infiltration (C) and interstitial fibrosis (D), expressed as percentage of total kidney area. (E) Categorical bubble plot showing the kidney expression levels of genes encoding for inflammatory cell markers (*Ptprc*, leukocytes; *Cd68*, macrophages, *Fut4*, neutrophils; *Cd19*, B cells; *Cd5*, T cells), chemokines (*Ccl2*, *Ccl12*, *Ccl5*), fibrosis markers (*Vim1*, *Tgfb1*, *Col1a1*, *Acta2*), and markers of tubular damage (*Lcn2* and *Havcr1*). Transcript levels were analyzed by real-time RT-qPCR. Bubble size is proportional to fold change relative to AL Tg^*Umod*wt^ mice. Bubble color corresponds to Log_10_ of the adjusted *P* value relative to AL Tg^*Umod*wt^ (one-way ANOVA followed by Dunnett multiple comparisons *post hoc* test). A Log_10_ adjusted *P* value ≤ −1.3 (white dashed line) was considered statistically significant. Data used to generate the categorical bubble plot are presented in Supplemental Table 2. (F) Immunofluorescence staining for transgenic UMOD (HA, in red) and LCN2 (in green) in kidney sections from AL or calorie restriction Tg^*Umod*wt^ and Tg^*Umod*C147W^ mice. The magnified field is indicated by a square on each picture. Scale bar: 40 *μ*m. Histograms represent quantification of the LCN2-positive signal in total kidney area or in TAL tubules (HA^+^). (G and H) Western blot analysis and quantification of LCN2 levels in kidney (G) and urine (uLCN2) (H). For kidney lysates, GAPDH was used as a loading control. Urine samples were normalized over total urine volume collected in 16 hours. *n*=5–6 mice/group (all females). Bars represent mean±SEM (in B–D and F) or ±SD (in G and H). (G and H) Data are reported as fold change relative to AL Tg^*Umod*wt^. Group comparisons were performed by one-way (in B–D, G, and H) or two-way (in F) ANOVA followed by Tukey correction. BF, bright field; LCN2, lipocalin-2; PL, polarized light; TAL, thick ascending limb of Henle's loop; uLCN2, urinary LCN2.

We then analyzed inflammation and fibrosis, two important ADTKD features. As expected, all histologic parameters of kidney damage (tubular dilation, inflammatory cell infiltration, and interstitial fibrosis) were increased in AL Tg^*Umod*C147W^ mice and reduced to levels comparable to Tg^*Umod*wt^ mice after calorie restriction (Figure 2, A–D, and Supplemental Figure 2, A and B). A similar trend was observed for kidney expression of inflammation- and fibrosis-related genes (Figure 2E and Supplemental Figure 2C and Supplemental Table 2). Calorie restriction reduced the expression of some genes related to inflammation (*i.e*., *Ptprc*, *Ccl5*) also in kidneys of Tg^*Umod*wt^ mice, substantiating the already described impact of calorie restriction on inflammatory cell metabolism.^35,36^

Tubular damage is an early event occurring in Tg^*Umod*C147W^ mice. Lipocalin-2 (LCN2), expressed in injured TECs,^37^ is induced as early as 1 week and remains highly expressed in adults, representing one of the most up-regulated genes identified in kidney transcriptional profiling.^14,24^ As the disease progresses, *Havcr1* (encoding kidney injury molecule-1)^38^ is also induced starting at 4 weeks.^24^ After calorie restriction, *Lcn2* and *Havcr1* expression levels were significantly reduced in kidneys of Tg^*Umod*C147W^ mice compared with their AL counterpart (Figure 2E).

Immunofluorescence analysis revealed that LCN2 signal was specifically induced in mutant uromodulin-expressing cells and that it was dramatically reduced, along with mutant protein aggregates, in calorie-restricted Tg^*Umod*C147W^ mice (Figure 2F). This was reflected by significant decrease of LCN2 levels in kidneys (Figure 2G) and urines (Figure 2H), implying that reducing mutant uromodulin ER retention rescues TAL cell damage in Tg^*Umod*C147W^ mice.

These results demonstrate that calorie restriction effectively ameliorates ADTKD-*UMOD* phenotype by reducing inflammation and fibrosis and rescuing tubular damage.

### Calorie Restriction Restored Autophagy in Mutant Uromodulin-Expressing Cells

Since reduced mutant uromodulin accumulation in kidneys of calorie-restricted mice was not explained by decreased gene expression or increased secretion, we hypothesized that it could be due to enhanced protein degradation. To gain mechanistic insight, we evaluated markers of autophagy. Induction of this pathway contributes to mutant uromodulin degradation in cell models^14^ and improves disease phenotype *in vivo*.^22^ Moreover, calorie restriction is reported to stimulate autophagy by suppressing mammalian target of rapamycin (mTOR) signaling, thus regulating cell catabolism.^39^

**Figure 3:**
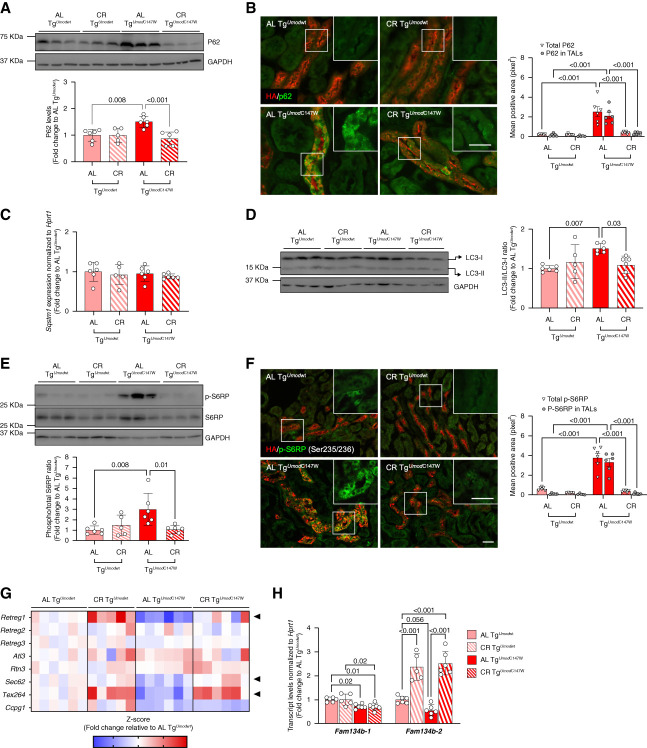
**Calorie restriction restored autophagy in mutant uromodulin-expressing cells.** (A) Western blot analysis and quantification of P62 kidney levels. GAPDH is reported as a loading control. (B) Immunofluorescence staining for P62 (in green) and transgenic UMOD (HA, in red) in kidney sections. The magnified field is indicated by a square on each picture. Scale bar: 20 *μ*m. Histograms represent quantification of the P62-positive signal in total kidney area or in TAL tubules (HA^+^). (C) Analysis of *Sqstm1* (encoding P62) kidney expression levels. (D) Western blot analysis of LC3 (autophagosome marker) and quantification of LC3-II/LC3-I (active/inactive) ratio. (E) Western blot analysis of phosphorylated (p-S6RP; Ser235/236) and total S6RP (downstream target of mTOR activity) in total kidney lysates. The graph shows the phosphorylated/total S6RP ratio. GAPDH is reported as a loading control. (F) Immunofluorescence staining for transgenic UMOD (HA, in red) and p-S6RP (in green) in kidney sections. The magnified field is indicated by a square on each picture. Scale bar: 20 *μ*m. Histograms represent quantification of the p-S6RP–positive signal in total kidney or in TAL tubules (HA^+^). (G) Heatmap showing the kidney expression levels of ER-phagy receptor genes *Retreg1* (encoding Fam134b), *Retreg2* (Fam134a), *Retreg3* (Fam134c), *Atl3*, *Rtn3*, *Sec62*, *Tex264*, and *Ccpg1* in AL or calorie restriction Tg^*Umod*wt^ and Tg^*Umod*C147W^ mice. Transcript levels were analyzed by real-time RT-qPCR. For each gene, expression levels are reported as *z*-score of the fold change relative to AL Tg^*Umod*wt^. Arrow heads indicate genes that are significantly downregulated in kidneys of AL Tg^*Umod*C147W^ mice compared with AL Tg^*Umod*wt^ mice and whose expression level is restored after calorie restriction. For all the analyzed genes, the fold change relative to AL Tg^*Umod*wt^ mice and statistical analysis is presented in Supplemental Table 3. (H) Transcript levels of *Fam134b-1* (full length) and *Fam134b-2* (*N*-terminal truncated, starvation-induced *Fam134b* isoform) in kidneys of AL or calorie restriction Tg^*Umod*wt^ and Tg^*Umod*C147W^ mice. *n*=5–6 mice/group (all females). Bars represent mean±SD (in A, C–E, and H) or ±SEM (in B and F). (A, C–E, and H) Data are reported as fold change relative to AL Tg^*Umod*wt^. Group comparisons were performed by one-way (in A, D, E, and H) or two-way (in B and F) ANOVA followed by Tukey correction. mTOR, mammalian target of rapamycin; S6RP, ribosomal protein S6.

We tested the expression of P62, an autophagy receptor that is itself degraded along with the substrate, thus representing a readout of autophagic flux.^40^ In kidneys of AL Tg^*Umod*C147W^ mice, we observed increased P62 protein levels, and specific accumulation of P62-positive punctae in cells expressing mutant uromodulin (Figure 3, A and B). This was associated with comparable levels of transcript (*Sqstm1*) in kidneys of mutant mice compared with Tg^*Umod*wt^ mice (Figure 3C), supporting reduced/impaired autophagic flux^41^ in mutant uromodulin-expressing cells, consistent with results in other ADTKD-*UMOD* models.^14,19,22^ Noteworthy, accumulation of P62 punctae in TALs as well as its kidney levels were strongly reduced in calorie-restricted Tg^*Umod*C147W^ mice (Figure 3, A and B), despite stable transcript levels (Figure 3C), suggesting rescue of autophagic flux. This was also demonstrated by kidney levels of LC3-II/LC3-I ratio, which are increased in AL Tg^*Umod*C147W^ mice and reduced to levels comparable to Tg^*Umod*wt^ mice after calorie restriction (Figure 3D).

To assess the potential involvement of mTOR pathway in the induction of autophagy upon calorie restriction, we measured phosphorylation of its downstream target, ribosomal protein S6 (S6RP). Compared with Tg^*Umod*wt^ mice, AL Tg^*Umod*C147W^ mice show increased kidney levels of phospho-S6RP that are almost exclusively observed in mutant uromodulin-expressing cells. Notably, the levels of S6RP were essentially normalized by calorie restriction (Figure 3, E and F).

Given the reduction of ER-retained mutant uromodulin on calorie restriction, we investigated ER-phagy, a form of autophagy mediated by selective receptors, that specifically degrades portions of the ER.^42^ We tested the kidney expression levels of genes encoding for some of the ER-phagy receptors so far reported in mammals.^43^ Among these receptors *Retreg1*, *Sec62,* and *Tex264* were downregulated in kidneys of mutant mice in basal conditions and their expression was restored to Tg^*Umod*wt^ levels after calorie restriction (Figure 3G and Supplemental Table 3). Since an *N*-terminal truncated isoform of FAM134B (FAM134B-2) encoded by *Retreg1* has been demonstrated to be transcriptionally enhanced to regulate starvation-induced ER-phagy,^44–46^ we specifically analyzed the expression of the different FAM134B isoforms. Both *Fam134b-1* (encoding the full-length protein) and *Fam134b-2* were downregulated in kidneys of AL Tg^*Umod*C147W^ mice, whereas only *Fam134b-2* was upregulated in kidneys of calorie-restricted Tg^*Umod*C147W^ mice (Figure 3H), supporting a role for calorie restriction in recovering ER-phagy in kidneys of Tg^*Umod*C147W^ mice.

These data strongly suggest that, by inhibiting mTOR overactivation in kidneys of mice expressing mutant uromodulin, calorie restriction rescues autophagy (and likely ER-phagy) in TAL cells, thus allowing degradation of ER-retained mutant uromodulin.

### Calorie Restriction Largely Reverted ADTKD Phenotype at Early Disease Stage

**Figure 4:**
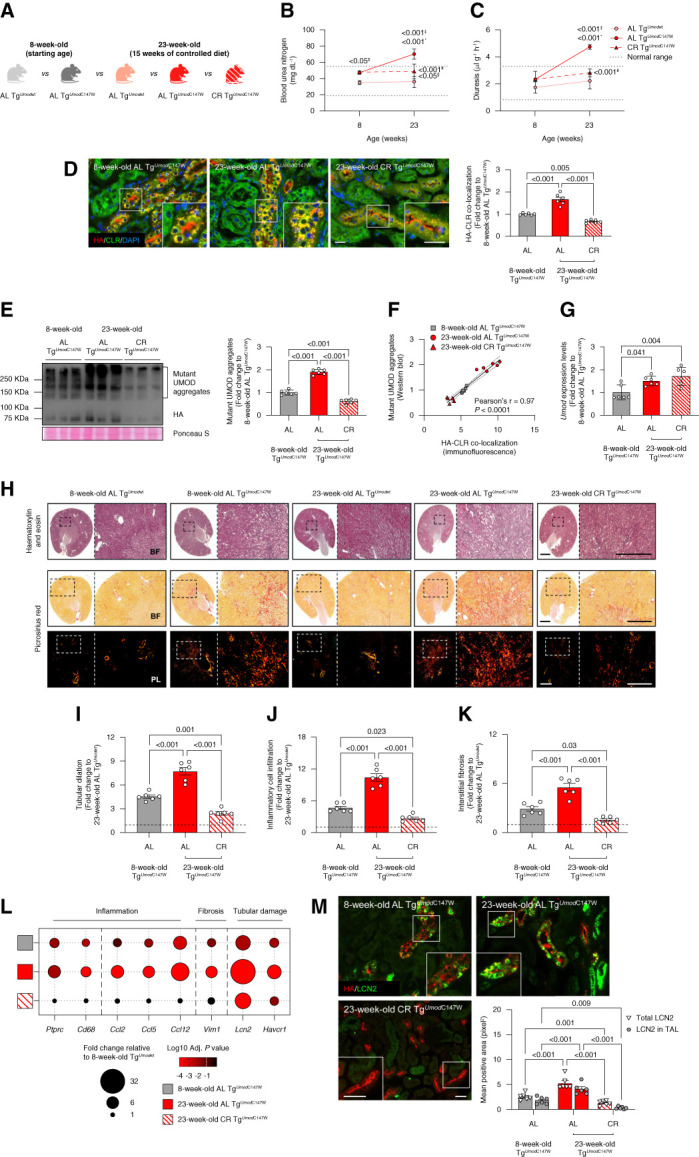
**Calorie restriction prevented ADTKD progression and largely reverted disease phenotype at early disease stage.** (A) To assess the potential of calorie restriction on ADTKD-*UMOD* phenotype reversal, 23-week-old AL Tg^*Umod*wt^ mice, AL or calorie restriction Tg^*Umod*C147W^ mice were compared with 8-week-old AL Tg^*Umod*wt^ and Tg^*Umod*C147W^ mice, corresponding to the age of mice at the beginning of the dietary intervention. (B and C) BUN (B) and diuresis (C) levels of mice at 8 weeks and 23 weeks of age. Gray dashed lines represent normal range values. ^$^*P* 8-week-old AL Tg^*Umod*wt^ versus 8-week-old AL Tg^*Umod*C147W^; ^*^P 23-week-old AL Tg^*Umod*wt^ versus 23-week-old AL Tg^*Umod*C147W^; ^#^*P* 23-week-old AL Tg^*Umod*C147W^ versus 23-week-old calorie restriction Tg^*Umod*C147W^; ^§^*P* 23-week-old AL Tg^*Umod*wt^ versus 23-week-old calorie restriction Tg^*Umod*C147W^; ^‡^*P* 8-week-old AL Tg^*Umod*C147W^ versus 23-week-old AL Tg^*Umod*C147W^. (D) Immunofluorescence staining for transgenic UMOD (HA, in red) and CLR (in green). Nuclei are counterstained with DAPI (in blue). Scale bar: 40 *μ*m. Histograms represent quantification of transgenic UMOD-CLR colocalization area. (E) Western blot in nonreducing conditions to detect transgenic UMOD (HA) in kidney lysates. Ponceau S staining is reported as a loading control. Graph represents quantification of HMW mutant UMOD aggregates. (F) Linear regression illustrating the correlation between mutant UMOD aggregates measured by Western blot and HA-CLR signal colocalization measured by immunofluorescence. The dotted lines show the 95% confidence intervals. Dots represent individual animals. (G) *Umod* transcript levels measured by real-time RT-qPCR. (H) Representative histology images of kidney sections stained with hematoxylin and eosin or picrosirius red and viewed in bright field (upper panels) or under polarized light (bottom panels). Scale bars: 2 mm and 600 *μ*m. (I–K) Quantification of tubular dilation (I), inflammatory cell infiltrate (J), and interstitial fibrosis (K). (L) Categorical bubble plot showing the kidney expression levels of inflammation- (*Ptprc*, *Cd68*, *Ccl2*, *Ccl5*, *Ccl12*), fibrosis- (*Vim1*), and tubular damage– (*Lcn2* and *Havcr1*) related genes. Transcript levels were analyzed by real-time RT-qPCR. Bubble size is proportional to fold change relative to 8-week-old AL Tg^*Umod*wt^. Bubble color corresponds to Log_10_ of the adjusted *P* value relative to 8-week-old AL Tg^*Umod*wt^ mice (one-way ANOVA, followed by Dunnett multiple comparisons *post hoc* test). A Log_10_ adjusted *P* value ≤ −1.3 (white dashed line) was considered statistically significant. Data used to generate the categorical bubble plot are presented in Supplemental Table 6. (M) Representative immunofluorescence staining for transgenic UMOD (HA, in red) and LCN2 (in green) in kidney sections. The magnified field is indicated by a square on each picture. Scale bar: 40 *μ*m. Histograms represent quantification of the LCN2-positive signal in total kidney area or in TAL tubules (HA^+^). *n*=6 mice/group (all females). Bars represent mean±SD (in B, C, E, and G) or ±SEM (in D, I–K, and M). Data are reported as fold change relative to 8-week-old AL Tg^*Umod*C147W^ (in D, E, and G) or to 8-week-old AL Tg^*Umod*wt^ (dashed line in I–K). Group comparisons were performed by one-way ANOVA, followed by Šidák (in B and C) or Tukey (in D, E, G, and I–K) correction, or two-way ANOVA, followed by Tukey correction (in M). ADTKD, autosomal dominant tubulointerstitial kidney disease; ADTKD-*UMOD*, *UMOD*-related autosomal dominant tubulointerstitial kidney disease.

Next, we assessed the effect of calorie restriction on disease progression by comparing 23-week-old AL and calorie-restricted Tg^*Umod*C147W^ mice with 8-week-old AL Tg^*Umod*C147W^ mice, corresponding to the age of animals at the beginning of treatment (Figure 4A). To evaluate kidney function, we performed plasma and urine biochemistry. At 23 weeks, AL Tg^*Umod*C147W^ mice developed significant polyuria and signs of kidney failure, as demonstrated by rise of BUN and diuresis levels (Figure 4, B and C, and Supplemental Tables 4 and 5). These parameters remained stable and within normal range in calorie-restricted Tg^*Umod*C147W^ mice.

The variation of these parameters was not an effect of calorie restriction *per se*, as they remained unchanged in calorie-restricted Tg^*Umod*wt^ mice compared with their AL counterpart (Supplemental Tables 4 and 5). These results demonstrate that calorie restriction prevents decline of kidney function in Tg^*Umod*C147W^ mice. ER accumulation of mutant uromodulin was already evident in 8-week-old Tg^*Umod*C147W^ mice (Figure 4, D–F), and the extent of ER retention significantly increased at 23 weeks, indicating disease progression. By contrast, in kidneys of 23-week-old calorie-restricted Tg^*Umod*C147W^ mice, mutant uromodulin aggregates appeared reduced to levels even lower than those at 8 weeks (Figure 4, D–F). Such reduction was not due to changes in *Umod* expression, which in fact increased with age in Tg^*Umod*C147W^ mice, irrespective of their dietary regimen (Figure 4G). These data demonstrate that calorie restriction not only prevents mutant uromodulin accumulation but is also effective in promoting clearance of existing protein aggregates. In Tg^*Umod*C147W^ mice, disease progression is also evidenced by progressive increase of tubular dilation, inflammation, and interstitial fibrosis (Figure 4, H–L, and Supplemental Figure 3, A and B, and Supplemental Table 6) and elevated expression of tubular damage markers (Figure 4, L and M, and Supplemental Figure 3C). Notably, after calorie restriction, tubular dilation, macrophage infiltration, and extracellular matrix deposition in kidneys of 23-weeks-old Tg^*Umod*C147W^ mice were comparable with wild-type animals and significantly decreased compared with 8-week-old AL Tg^*Umod*C147W^ mice (Figure 4, H–L, and Supplemental Figure 3, A and B, and Supplemental Table 6). Moreover, calorie restriction strongly attenuated tubular damage, as demonstrated by significant reduction of LCN2 in TAL tubules of 23-week-old calorie-restricted Tg^*Umod*C147W^ mice compared with their 8-week-old genotype-matched counterpart (Figure 4M and Supplemental Figure 3C).

These data collectively demonstrate that, when started at early stage of disease, calorie restriction prevents tubular damage and disease progression and largely rescues the existing kidney injury in Tg^*Umod*C147W^ mice.

### Calorie Restriction Blocked Disease Progression in Mice with Advanced ADTKD-*UMOD*

**Figure 5:**
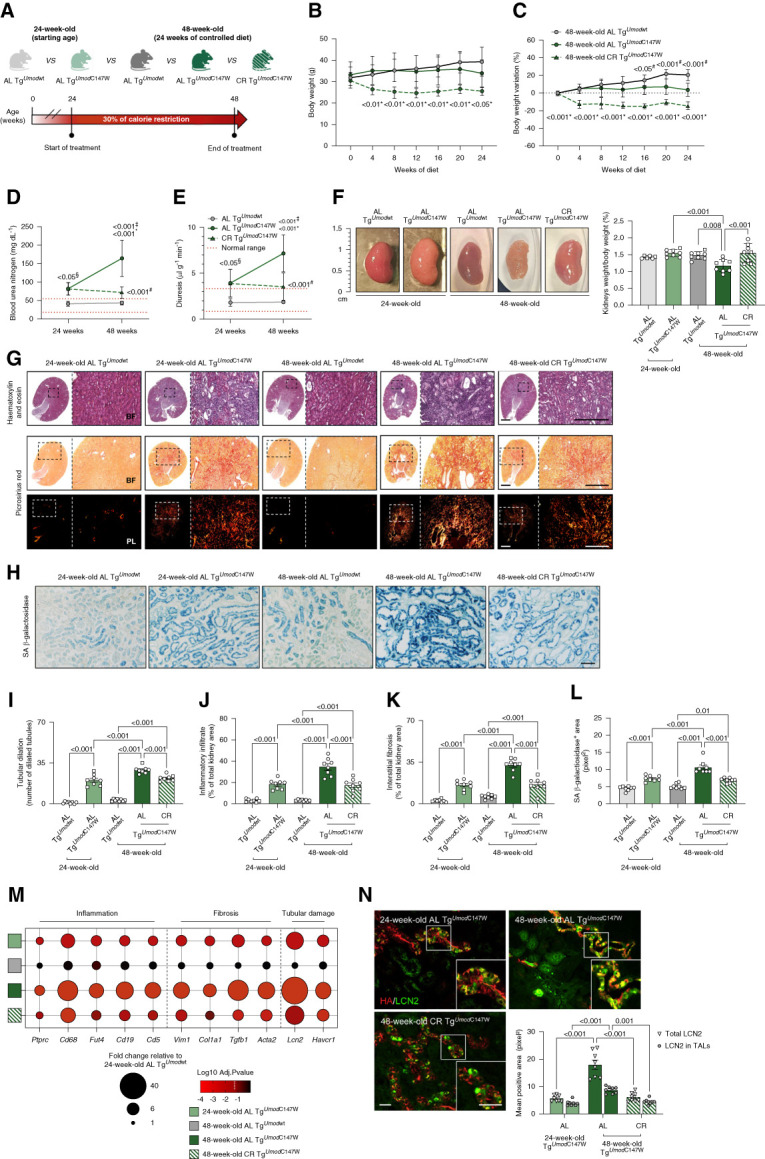
**Calorie restriction prevented ADTKD-*UMOD* progression at advanced disease stage.** (A) Schematic protocol of calorie restriction treatment at advanced disease stage. (B and C) Body weight (B) and percentage of weight loss compared with the initial time point (C) of mice during the diet. ^*^P 48-week-old AL Tg^*Umod*C147W^ versus 48-week-old calorie restriction Tg^*Umod*C147W^; ^#^*P* 48-week-old AL Tg^*Umod*wt^ versus 48-week-old AL Tg^*Umod*C147W^. (D and E) BUN (D) and diuresis (E) levels for the indicated experimental groups. Red dashed lines represent normal range values. ^§^*P* < 0.05 24-week-old AL Tg^*Umod*wt^ versus 24-week-old AL Tg^*Umod*C147W^; ^*^P 48-week-old AL Tg^*Umod*wt^ versus 48-week-old AL Tg^*Umod*C147W^; ^‡^*P* 24-week-old AL Tg^*Umod*C147W^ versus 48-week-old AL Tg^*Umod*C147W^; ^#^*P* 48-week-old AL Tg^*Umod*C147W^ versus 48-week-old calorie restriction Tg^*Umod*C147W^. (F) Representative picture of kidneys from 24-week-old AL Tg^*Umod*wt^ and Tg^*Umod*C147W^, 48-week-old AL Tg^*Umod*wt^ and 48-week-old AL or calorie restriction Tg^*Umod*C147W^ mice and quantification of the kidney/body weight index. *n*=8 mice/group (five female, three male). (G) Representative histology images of kidney sections stained with hematoxylin and eosin or picrosirius red and viewed in bright field (upper panels) or under polarized light (bottom panels). Scale bars: 2 mm and 600 *μ*m. (H) Representative images of SA *β*-galactosidase (SA *β*-gal) staining of kidney sections. Scale bar: 500 *μ*m. (I–L) Quantification of tubular dilation (I), inflammatory cell infiltrate (J), interstitial fibrosis (K), and SA *β*-gal–positive areas (L). (M) Categorical bubble plot showing the kidney expression levels of inflammation- (*Ptprc*, *Cd68*, *Fut4*, *Cd19*, *Cd5*), fibrosis- (*Vim1*, *Col1a1*, *Tgfb1*, *Acta2*), and tubular damage– (*Lcn2*, *Havcr1*) related genes. Transcript levels were analyzed by real-time RT-qPCR. Bubble size is proportional to fold change relative to 24-week-old AL Tg^*Umod*wt^ mice; bubble color corresponds to Log_10_ of the adjusted *P* value compared with 24-week-old AL Tg^*Umod*wt^ mice (one-way ANOVA, followed by Dunnett multiple comparisons *post hoc* test). A Log_10_ adjusted *P* value ≤ −1.3 (white dashed line) was considered statistically significant. Data used to generate the categorical bubble plot are presented in Supplemental Table 9. (N) Immunofluorescence staining for transgenic UMOD (HA, in red) and LCN2 (in green) in kidney sections. The magnified field is indicated by a square on each picture. Scale bar: 40 *μ*m. Histograms represent quantification of the LCN2-positive signal in total kidney area or in TAL tubules (HA^+^). *n*=8 mice/group (five females, three males). Data are reported as mean±SD (in B–F) or ±SEM (in I–N). Group comparisons were performed by using one-way ANOVA, followed by Šídák correction (in F and I–L), or two-way ANOVA, followed by Tukey correction (in N). SA, senescence-associated.

To substantiate the translational relevance of calorie restriction, we then investigated its effectiveness at an advanced disease stage. Tg^*Umod*C147W^ mice were fed a 30% calorie restriction diet for 24 weeks, starting at 24 weeks. To evaluate the potential of calorie restriction in ameliorating disease phenotype, 48-week-old calorie-restricted Tg^*Umod*C147W^ mice were compared with 24-week-old and 48-week-old AL Tg^*Umod*C147W^ and Tg^*Umod*wt^ mice (Figure 5A).

The prolonged calorie restriction protocol led to a relatively mild reduction of mice body weight (about 15%; Figure 5, B and C). As expected, 24-week-old AL Tg^*Umod*C147W^ mice showed already compromised kidney function that significantly worsened with aging. Instead, such drop of kidney function is not observed in 48-week-old calorie-restricted Tg^*Umod*C147W^ mice showing levels of plasma creatinine and BUN, urinary creatinine, and diuresis comparable with 24-week-old mutant mice (Figure 5, D and E, and Supplemental Tables 7 and 8). The beneficial effect of calorie restriction was also evident when comparing kidney morphology at the end of treatment as the pale, shrunk appearance of kidneys of AL Tg^*Umod*C147W^ mice, resembling patient small-sized kidneys,^47^ was remarkably rescued (Figure 5F). In line with kidney function and morphology, we observed progressive worsening of disease parameters in 48-week-old AL Tg^*Umod*C147W^ mice compared with their 24-week-old counterpart that was essentially blocked by calorie restriction. This was observed for all investigated markers of inflammation, fibrosis, and kidney damage, as assessed by histologic (Figure 5, G and I–K), immunofluorescence (Supplemental Figure 4), and gene expression analyses (Figure 5M and Supplemental Table 9). Since this experiment included aged mice, we also evaluated the kidney activity of senescence-associated *β*-galactosidase (SA *β*-gal), a predictor of kidney outcome in patients with CKD of different origin.^48^ Interestingly, senescence was already increased in 24-week-old Tg^*Umod*C147W^ mice, relative to Tg^*Umod*wt^ mice. Calorie restriction was effective in blocking further increase of senescence that was instead observed in 48-week-old AL Tg^*Umod*C147W^ mice (Figure 5, H and L).

We also evaluated the effect of diet on preserving TAL integrity. Compared with 24-week-old AL Tg^*Umod*C147W^ mice, 48-week-old AL Tg^*Umod*C147W^ mice show altered morphology of TAL segments and decreased expression the Na^+^-K^+^-2Cl^−^ cotransporter (NKCC2, marker of TAL cells) (Supplemental Figure 5, A–E) and significant increase of LCN2 levels (Figure 5N and Supplemental Figure 5F). All these parameters suggest progressive cell damage and possibly TAL cell loss in kidneys of 48-week-old AL Tg^*Umod*C147W^ mice, likely explaining why we did not observe further increase of mutant uromodulin aggregates compared with 24-week-old AL Tg^*Umod*C147W^ mice (Supplemental Figure 5A). Importantly, in 48-week-old calorie-restricted Tg^*Umod*C147W^ mice, we observed preserved TAL morphology (Supplemental Figure 5C), rescued expression levels of NKCC2 (Supplemental Figure 5, D and E), and reduced LCN2 expression (Figure 5N and Supplemental Figure 5F), suggesting a role of calorie restriction in preventing further TAL damage in Tg^*Umod*C147W^ mice.

In sum, our data demonstrate that initiating calorie restriction at a later stage of disease can still be effective in blocking ADTKD-*UMOD* progression.

### Unsupervised Multivariable Analysis of the Overall Therapeutic Effect of Calorie Restriction

**Figure 6:**
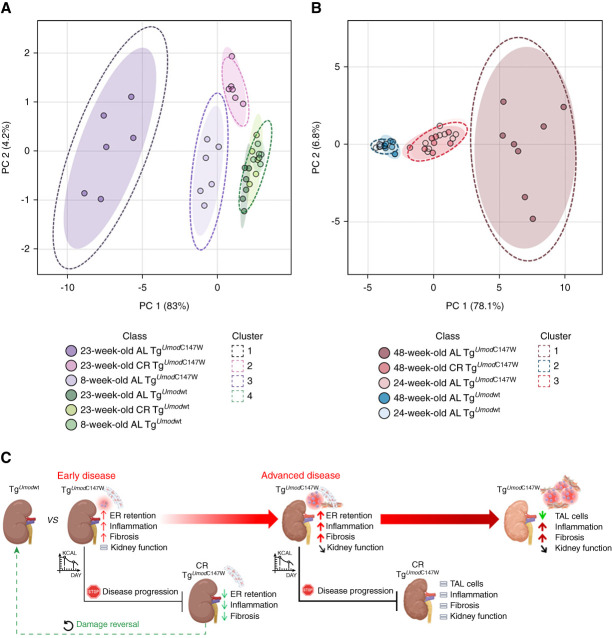
**Summary of the effect of calorie restriction at early and advanced ADTKD-*UMOD* stages.** (A) PCA performed using 15 parameters measured in 8-week-old AL Tg^*Umod*wt^ or Tg^*Umod*C147W^ mice and 23-week-old AL or calorie restriction Tg^*Umod*wt^ and Tg^*Umod*C147W^ mice. A heatmap of the parameters used for the analysis is shown in Supplemental Figure 6A. F-value: 148.19; *R*-squared: 0.96; *P* < 0.001 (based on 999 permutations; PERMANOVA). K-means clustering of samples revealed four distinct clusters: cluster 1 (in black) is formed by 23-week-old AL Tg^*Umod*C147W^ mice, cluster 2 (in pink) is formed by 23-week-old calorie restriction Tg^*Umod*C147W^ mice, cluster 3 (in purple) is formed by 8-week-old AL Tg^*Umod*C147W^ mice, while 8-week-old AL Tg^*Umod*wt^, 23-week-old AL Tg^*Umod*wt^ and 23-week-old calorie restriction Tg^*Umod*wt^ mice are all included in cluster 4 (in green). *n*=6 mice/group (all females). (B) PCA performed using 21 parameters measured in 24-week-old AL Tg^*Umod*wt^ or Tg^*Umod*C147W^, 48-week-old AL Tg^*Umod*wt^ and 48-week-old AL or calorie restriction Tg^*Umod*C147W^ mice. A heatmap of the parameters used for the analysis is shown in Supplemental Figure 6B. F-value: 63.45; *R*-squared: 0.87; *P* < 0.001 (based on 999 permutations; PERMANOVA). K-means clustering of samples, based on the same disease parameters, revealed three distinct clusters: cluster 1 (in brown) is formed by 48-week-old AL Tg^*Umod*C147W^ mice, cluster 2 (in blue) is formed by 24-week-old AL and 48-week-old AL Tg^*Umod*wt^ mice, and cluster 3 (in red) is formed by 24-week-old AL Tg^*Umod*C147W^ and 48-week-old calorie restriction Tg^*Umod*C147W^ mice. *n*=8 mice/group. Each point represents an individual animal, color-coded according to its experimental group. Ellipses represent the 95% CIs area of each group. Dashed ellipses indicate 95% CIs for each cluster centroid. (C) Schematic representation summarizing the phenotypic effects induced by calorie restriction in Tg^*Umod*C147W^ mice at early and advanced disease stages. CI, confidence interval; PCA, principal component analysis; PERMANOVA, permutational multivariate analysis of variance.

Finally, to integrate the values obtained for the characterization of the effect of calorie restriction on ADTKD-*UMOD* onset and progression, we performed unsupervised multivariable analysis. PCA followed by K-means clustering was conducted using 15 disease parameters evaluated at early stage of disease (Figure 6A). PCA highlighted a very strong separation between all experimental groups. K-means analysis revealed separation of 8-week-old AL Tg^*Umod*C147W^ and 23-week-old AL Tg^*Umod*C147W^ groups into two distinct clusters (clusters 1 and 3, respectively), consistent with the progression of disease severity with age. Instead, all Tg^*Umod*wt^ groups formed a single cluster (cluster 4), clearly separated from the previous two. Notably, 23-week-old calorie-restricted Tg^*Umod*C147W^ mice formed a fourth cluster (cluster 2), distinct from the other clusters composed by mutant mice, and more similar to wild-type animals (Figure 6A and Supplemental Figure 6A). This analysis substantiates the experimental evidence that, at early disease stages, calorie restriction blocks ADTKD-*UMOD* progression and considerably ameliorates the already established kidney damage.

A similar analysis was performed on the 21 parameters tested at advanced disease stage. PCA analysis demonstrated a strong difference between groups (Figure 6B). Both PCA and K-means analyses revealed that wild-type mice cluster together, irrespectively of their age (24-week-old and 48-week-old AL Tg^*Umod*wt^ mice, cluster 2) and were clearly separated from 48-week-old AL Tg^*Umod*C147W^ mice (cluster 1). Instead, 48-week-old calorie-restricted Tg^*Umod*C147W^ mice grouped together with 24-week-old AL Tg^*Umod*C147W^ mice (cluster 3) and were more similar to wild-type mice than their AL counterpart (Figure 6B and Supplemental Figure 6B). This reflects the experimental observations that calorie restriction prevents age-associated worsening of disease parameters.

Overall, these analyses provide additional and unsupervised evidence supporting the remarkable therapeutic efficacy of calorie restriction at both early and advanced ADTKD-*UMOD* stages (Figure 6C).

## Discussion

This study demonstrates the effectiveness of calorie restriction in reducing the intracellular load of mutant uromodulin and ameliorating kidney damage in ADTKD-*UMOD*. At early disease stage, calorie restriction prevented inflammation and progressive decline of kidney function, rescued fibrosis, and largely reverted the already established phenotype in Tg^*Umod*C147W^ mice. At advanced disease stage, calorie restriction was effective in blocking disease progression and worsening of kidney function. This beneficial effect is likely due to the action of calorie restriction in restoring autophagy in TECs expressing mutant uromodulin, in inducing degradation of ER-retained uromodulin, and in reducing inflammation.

Interventions aimed at reducing mutant uromodulin intracellular load could represent a valuable therapeutic option for ADTKD-*UMOD*, given the shared effect of *UMOD* mutations. Recently, a strategy aimed at rescuing mutant uromodulin trafficking, increasing its secretion, was shown to ameliorate kidney damage in an ADTKD-*UMOD* mouse model.^25^ However, the possible consequences of long-term secretion of mutant uromodulin have not been investigated. Here, we instead used a strategy aimed at degrading mutant uromodulin through induction of autophagy prompted by calorie restriction. Calorie restriction is described as the most powerful nongenetic intervention that delays age-associated pathologies and extends lifespan across multiple species^26^ by acting on multiple molecular mechanisms, including enhancement of proteostasis, modulation of nutrient-sensing,^29^ and autophagy induction. Its effect on mutant uromodulin degradation is demonstrated by remarkable reduction of uromodulin protein level, in the absence of any effect on gene expression or urinary secretion. Consistent with other studies,^14,19,22^ we reported the presence of P62 punctae in TALs and increased LC3-II/LC3-I ratio in kidneys of Tg^*Umod*C147W^ mice. This is likely downstream of mTOR activation, as demonstrated by increased levels of phospho-S6RP, suggesting defective autophagy in cells expressing mutant uromodulin. After calorie restriction, P62 punctae and phospho-S6RP were strongly decreased in TALs, along with ER-retained mutant uromodulin. This indicates that the beneficial effect of calorie restriction is likely mediated by quenching of mTOR overactivation, a mechanism previously described in mouse models of autosomal dominant polycystic kidney disease (ADPKD) under mild to moderate (10%–40%) calorie restriction.^49^ In Tg^*Umod*C147W^ mice, calorie restriction restored autophagy in TALs and promoted clearance of mutant uromodulin. Our findings are consistent with evidence that stimulating autophagy leads to intracellular removal of mutant uromodulin in cells^14,19^ and that its induction in two different ADTKD-*UMOD* mouse models ameliorates disease phenotype.^22^ We hypothesize that clearance of mutant uromodulin aggregates is mediated by ER-phagy^50^ rather than bulk autophagy. In kidneys of Tg^*Umod*C147W^ mice, the ER-phagy receptors *Retreg1*, *Sec62,* and *Tex264* were downregulated, suggesting impaired ER-phagy and defective clearance of ER-retained uromodulin. After calorie restriction, their transcript levels were recovered. *Retreg1* encodes FAM134B, a key regulator of ER turnover.^51^ Its expression is tightly controlled by the nutrient-sensitive SESTRIN2–mTORC1–TFEB/TFE3 axis^44,52^ and, under ER stress, FAM134B scavenges ERAD-resistant misfolded clients interacting with the ER chaperone calnexin.^53,54^ An N-terminal-truncated isoform of FAM134B (*Fam134b-2*) is transcriptionally induced to drive ER-phagy under nutrient depletion.^45^ This is confirmed in our models as *Fam134b-2*, but not full-length *Fam134b-1*, was induced by calorie restriction. TEX264 was implicated in ER remodeling during nutrient stress.^55^ SEC62, a translocon component, aids the autophagic degradation of the ER membranes during recovery from ER stress.^56^ Further studies are needed to substantiate the causal link between autophagy induction and the observed beneficial effect of calorie restriction (*e.g*., by assessing the effect of the dietary intervention under genetic or pharmacologic inhibition of autophagy) and the role of ER-phagy in mutant uromodulin degradation. Regarding ADTKD-*UMOD* as a paradigm for ER storage disease, our findings could have broader relevance, although the effect of calorie restriction on models of other proteinopathies has to be tested.

Calorie restriction also ameliorated kidney inflammation, an effect that could be an indirect consequence of reduced TAL stress, following reduction of ER-retained mutant uromodulin. However, a synergistic effect due to the direct anti-inflammatory role of calorie restriction seems likely. Several studies indeed demonstrated the potent capability of calorie restriction in modulating inflammation in different settings,^27,57^ due to its direct impact on inflammatory cell metabolism.^35,36^ Calorie restriction also had a remarkable impact on kidney fibrosis that is likely downstream of its anti-inflammatory effect. Beyond these kidney-specific effects, it remains to be explored if systemic effects improving the overall metabolic state and BP^58^^–^^60^ could contribute to the observed amelioration of disease phenotype in Tg^*Umod*C147W^ mice.

In 23-week-old calorie-restricted Tg^*Umod*C147W^ mice, inflammation and fibrosis markers were reduced to levels even lower than those at the start of the diet and were comparable with wild-type mice, demonstrating that at early disease stages, calorie restriction not only blocked progression, but it also markedly ameliorated the already established kidney damage. Whether the repair of kidney damage in Tg^*Umod*C147W^ mice is induced by nephrogenic pathways, similar to what recently described in a glomerulopathy model administered fasting-mimicking diet,^61^ remains to be tested. Kidney plasticity and the ability to restore structural and functional alteration have been described in a mouse model of ADPKD in which re-expression of *Pkd* genes reversed disease phenotype. Recovery was complete when re-expression occurred at early stage of disease, while it was partial when induced at later time points.^62^ Similarly, we showed that calorie restriction was still effective in preventing disease progression even when initiated at advanced disease stage. Considering the relatively late onset of kidney failure in ADTKD-*UMOD*, this finding could be relevant for patient management as it might delay the need of replacement therapies.

In 48-week-old AL Tg^*Umod*C147W^ mice, we observed a marked reduction of NKCC2 expression with most TAL tubules showing dilation, flattened epithelia, and increased LCN2 expression. This likely reflects progressive TAL cell damage and loss that we described to start at 24 weeks.^11^ By contrast, calorie restriction reduced LCN2 levels and maintained the typical cuboidal morphology of TAL cells and NKCC2 expression, suggesting that it preserved the integrity of uromodulin-expressing cells and mitigated tubular damage. In 48-week-old Tg^*Umod*C147W^ kidneys, we also reported increased activity of the SA *β*-gal, a well-established marker of cell senescence.^63^ Senescence was described as a predictor of kidney outcome in patients with CKD of diverse etiology and a factor exacerbating inflammation and fibrosis.^48,64–66^ Calorie restriction can delay senescence and improve senescence-associated changes in the kidney.^67,68^ Consistently, we observed a strong reduction of SA *β*-gal activity in kidneys of calorie-restricted Tg^*Umod*C147W^ mice, suggesting an effect on senescence. Further investigations are needed to clarify the relevance of senescence in ADTKD-*UMOD* pathogenesis and its potential as therapeutic target.

Clinical trials investigating calorie restriction as an adjuvant therapy are currently ongoing in different disease settings, including multiple sclerosis,^69^ ADPKD with overweight or obesity,^70^ and type 2 diabetes.^60^ A possible limitation of calorie restriction in clinical settings is patient adherence to dietary restriction and the difficulty for clinicians to objectively measure adherence.^71^ Moreover, considering the metabolic differences between mice and humans, it is possible that more conservative levels of calorie restriction are needed to ensure safety and physiologic relevance in clinical settings. Alternative strategies could use different dietary interventions, such as time-restricted feeding, intermittent fasting, or restriction of specific macronutrients^72–74^ or calorie restriction mimetics^75^ that could increase patient adherence to therapy. Additional studies are required to identify end points and biomarkers to transfer calorie restriction or similar strategies to ADTKD-*UMOD* patients.

In conclusion, this work demonstrates that calorie restriction could be a valuable therapeutic intervention to treat ADTKD-*UMOD*.

## Supplementary Material

## Data Availability

Original data generated for the study are available in a public access repository. Data Type: Image Data; Observational Data. Repository Name: San Raffaele Open Research Data Repository (https://ordr.hsr.it/datasets/ry54nh5g2r/1).

## Declarative Statements

All animal experiments were conducted in accordance with the NIH Guide for the Care and Use of Laboratory Animals or an equivalent standard that meets or exceeds the ethical and welfare requirements outlined in the NIH Guide. All protocols were approved by the appropriate institutional animal care and use committee. This research was posted on a preprint server. https://doi.org/10.64898/2025.12.16.694693.

## Supplemental Material

Supplemental Methods.

Supplemental Figure 1. Effect of calorie restriction on uromodulin in male Tg^*Umod*C147W^ mice.

Supplemental Figure 2. Effect of calorie restriction on renal inflammation and fibrosis in male Tg^*Umod*C147W^ mice.

Supplemental Figure 3. Effect of calorie restriction on inflammation, fibrosis, and tubular damage in Tg^*Umod*C147W^ mice at early disease stage.

Supplemental Figure 4. Effect of calorie restriction on kidney inflammation and fibrosis in Tg^*Umod*C147W^ mice at advanced disease stage.

Supplemental Figure 5. Effect of calorie restriction on TAL cell integrity in Tg^*Umod*C147W^ mice at advanced disease stage.

Supplemental Figure 6. Heatmap of disease parameters tested to evaluate the effect of calorie restriction at early and advanced disease stage.

Supplemental Table 1. List and sequences of primers used for mouse genotyping.

Supplemental Table 2. Data used to generate the categorical bubble plot in Figure 2.

Supplemental Table 3. Kidney expression levels of genes encoding ER-phagy receptors.

Supplemental Table 4. BUN and serum creatinine levels in 8-week-old AL Tg^*Umod*wt^ and Tg^*Umod*C147W^ mice and 23-week-old AL or calorie-restricted Tg^*Umod*wt^ and Tg^*Umod*C147W^ mice.

Supplemental Table 5. Diuresis and urinary creatinine levels in 8-week-old AL Tg^*Umod*wt^ and Tg^*Umod*C147W^ mice and 23-week-old AL or calorie-restricted Tg^*Umod*wt^ and Tg^*Umod*C147W^ mice.

Supplemental Table 6. Data used to generate the categorical bubble plot in Figure 4.

Supplemental Table 7. BUN and serum creatinine levels in 24-week-old AL Tg^*Umod*wt^ and Tg^*Umod*C147W^, 48-week-old AL Tg^*Umod*wt^ and 48-week-old AL or calorie-restricted Tg^*Umod*C147W^ mice.

Supplemental Table 8. Diuresis and urinary creatinine levels in 24-week-old AL Tg^*Umod*wt^ and Tg^*Umod*C147W^, 48-week-old AL Tg^*Umod*wt^, and 48-week-old AL or calorie-restricted Tg^*Umod*C147W^ mice.

Supplemental Table 9. Data used to generate the categorical bubble plot in Figure 5.

Supplemental Table 10. List and sequences of primers used for gene expression analysis by SYBR Green real-time RT-qPCR.

Supplemental Table 11. List of antibodies used for Western blot and/or immunofluorescence analysis.
