# Supplementary material for: Calorie Restriction Leads to Degradation of Mutant Uromodulin and Ameliorates Inflammation and Fibrosis in UMOD-Related Kidney Disease
Source: J Am Soc Nephrol. 2026 Feb 3;37(8):1704–19. doi: 10.1681/ASN.0000001032 (PMC13406265; doi:10.1681/ASN.0000001032)
Supplement: Supplementary file 1 [file jasn-37-1704-s001.pdf]

ASN American Society of Nephrology JASN Journal of the American Society of Nephrology KIDNEY360 Accessing Our World From Every Angle CJASN Clinical Journal of the American Society of Nephrology

ASN Journal Disclosure Form

As per ASN journal policy, I have disclosed any financial relationships or commitments I have held in the past 36 months as included below. I have listed my Current Employer below to indicate there is a relationship requiring disclosure. If no relationship exists, my Current Employer is not listed.

B. Canciani has nothing to disclose.

I understand that the information above will be published within the journal article, if accepted, and that failure to comply and/or to accurately and completely report the potential financial conflicts of interest could lead to the following: 1) Prior to publication, article rejection, or 2) Post-publication, sanctions ranging from, but not limited to, issuing a correction, reporting the inaccurate information to the authors' institution, banning authors from submitting work to ASN journals for varying lengths of time, and/or retraction of the published work.

Name: Barbara Canciani
Manuscript ID: JASN-2025-001684
Manuscript Title: Calorie restriction leads to degradationof mutant uromodulin and ameliorates inflammation and fibrosis in UNOD-related kidney disease
Date of Completion: January 5, 2026
Disclosure Updated Date: January 5, 2026

ASN American Society of Nephrology · JASN Journal of the American Society of Nephrology · KIDNEY360 Accessing Our World From Every Angle · CJASN Clinical Journal of the American Society of Nephrology

ASN Journal Disclosure Form

As per ASN journal policy, I have disclosed any financial relationships or commitments I have held in the past 36 months as included below. I have listed my Current Employer below to indicate there is a relationship requiring disclosure. If no relationship exists, my Current Employer is not listed.

M. Cratere reports the following:
Employer: San Raffaele Scientific Institute

I understand that the information above will be published within the journal article, if accepted, and that failure to comply and/or to accurately and completely report the potential financial conflicts of interest could lead to the following: 1) Prior to publication, article rejection, or 2) Post-publication, sanctions ranging from, but not limited to, issuing a correction, reporting the inaccurate information to the authors' institution, banning authors from submitting work to ASN journals for varying lengths of time, and/or retraction of the published work.

Name: Mariapia Giuditta Cratere
Manuscript ID: JASN-2025-001684
Manuscript Title: Calorie restriction leads to degradation of mutant uromodulin and ameliorates inflammation and fibrosis in UMOD-related kidney disease
Date of Completion: January 4, 2026
Disclosure Updated Date: January 4, 2026

ASN American Society of Nephrology | JASN Journal of the American Society of Nephrology | KIDNEY360 Accessing Our World From Every Angle | CJASN Clinical Journal of the American Society of Nephrology

ASN Journal Disclosure Form

As per ASN journal policy, I have disclosed any financial relationships or commitments I have held in the past 36 months as included below. I have listed my Current Employer below to indicate there is a relationship requiring disclosure. If no relationship exists, my Current Employer is not listed.

B. Perrone reports the following:
Employer: San Raffaele Scientific Institute

I understand that the information above will be published within the journal article, if accepted, and that failure to comply and/or to accurately and completely report the potential financial conflicts of interest could lead to the following: 1) Prior to publication, article rejection, or 2) Post-publication, sanctions ranging from, but not limited to, issuing a correction, reporting the inaccurate information to the authors' institution, banning authors from submitting work to ASN journals for varying lengths of time, and/or retraction of the published work.

Name: Benedetta Perrone
Manuscript ID: JASN-2025-001684
Manuscript Title: Calorie restriction leads to degradation of mutant uromodulin and ameliorates inflammation and fibrosis in UMOD-related kidney disease
Date of Completion: January 5, 2026
Disclosure Updated Date: January 5, 2026

ASN American Society of Nephrology
JASN Journal of the American Society of Nephrology
KIDNEY360 Accessing Our World From Every Angle
CJASN Clinical Journal of the American Society of Nephrology

ASN Journal Disclosure Form

As per ASN journal policy, I have disclosed any financial relationships or commitments I have held in the past 36 months as included below. I have listed my Current Employer below to indicate there is a relationship requiring disclosure. If no relationship exists, my Current Employer is not listed.

L. Rampoldi reports the following:
Employer: San Raffaele Scientific Institute; Consultancy: Amicus Therapeutics; Research Funding: Amicus Therapeutics Investigator Initiated Program (year 2021); Honoraria: Amicus Therapeutics (speaker honoraria, advisory board); and Advisory or Leadership Role: Biorek s.r.l. (scientific advisory board); Amicus Therapeutics (scientific advisory board).

I understand that the information above will be published within the journal article, if accepted, and that failure to comply and/or to accurately and completely report the potential financial conflicts of interest could lead to the following: 1) Prior to publication, article rejection, or 2) Post-publication, sanctions ranging from, but not limited to, issuing a correction, reporting the inaccurate information to the authors' institution, banning authors from submitting work to ASN journals for varying lengths of time, and/or retraction of the published work.

Name: Luca Rampoldi
Manuscript ID: JASN-2025-001684
Manuscript Title: Calorie restriction leads to degradation of mutant uromodulin and ameliorates inflammation and fibrosis in UMOD-related kidney disease
Date of Completion: January 5, 2026
Disclosure Updated Date: January 5, 2026

ASN
American Society of Nephrology
JASN
Journal of the American Society of Nephrology
KIDNEY360
Accessing Our World From Every Angle
CJASN
Clinical Journal of the American Society of Nephrology

ASN Journal Disclosure Form

As per ASN journal policy, I have disclosed any financial relationships or commitments I have held in the past 36 months as included below. I have listed my Current Employer below to indicate there is a relationship requiring disclosure. If no relationship exists, my Current Employer is not listed.

C. Schaeffer reports the following:
Employer: San Raffaele Scientific Institute

I understand that the information above will be published within the journal article, if accepted, and that failure to comply and/or to accurately and completely report the potential financial conflicts of interest could lead to the following: 1) Prior to publication, article rejection, or 2) Post-publication, sanctions ranging from, but not limited to, issuing a correction, reporting the inaccurate information to the authors' institution, banning authors from submitting work to ASN journals for varying lengths of time, and/or retraction of the published work.

Name: Celine Schaeffer
Manuscript ID: JASN-2025-001684
Manuscript Title: Calorie restriction leads to degradation of mutant uromodulin and ameliorates inflammation and fibrosis in UMOD-related kidney disease
Date of Completion: January 2, 2026
Disclosure Updated Date: January 2, 2026
